# Comparative efficacy of half-dose and one-third-dose photodynamic therapy in chronic central serous chorioretinopathy: a retrospective study

**DOI:** 10.1186/s40942-025-00657-6

**Published:** 2025-03-18

**Authors:** Mohsen Farvardin, Dorna Eghtedari, Maryam Shahmohammadi, Mohammadkarim Johari

**Affiliations:** Poostchi Ophthalmology Research Center, Department of Ophthalmology, School of Medicine, Shiraz University of Medical Sciences, Shiraz, Iran

**Keywords:** Chronic central serous chorioretinopathy (CSC), Reduced-dose photodynamic therapy (PDT), Visual acuity improvement, Subretinal fluid resolution, Choroidal thickness changes

## Abstract

**Purpose:**

To compare the efficacy and safety of half-dose and one-third-dose photodynamic therapy (PDT) with verteporfin in patients with chronic central serous chorioretinopathy (CSC).

**Methods:**

This retrospective study included 72 eyes from 72 patients with chronic CSC treated with either one-third-dose (2 mg/m²) or half-dose (3 mg/m²) PDT. Best-corrected visual acuity (BCVA), central retinal thickness (CRT), subretinal fluid (SRF) thickness, subfoveal choroidal thickness (SFCT), and optical coherence tomography (OCT) features were evaluated at baseline, 3 months, and 12 months. Fluorescein angiography (FA) was used to guide laser application. Treatment outcomes, including SRF resolution, BCVA gain, and recurrence rates, were compared between the two groups.

**Results:**

At 12 months, complete SRF resolution was achieved in 40 eyes (78.4%) in the half-dose group and 15 eyes (71.4%) in the one-third-dose group. The recurrence rate of SRF was significantly higher in the one-third-dose group (20%) compared to the half-dose group (7.5%) (*P* =.015). BCVA improved significantly in both groups, with mean increases from 72.4 ± 3.9 to 77.1 ± 5.6 letters in the one-third-dose group and from 74.4 ± 4.2 to 80.2 ± 2.19 letters in the half-dose group. The proportion of patients achieving a ≥ 10-letter gain was higher in the half-dose group (52%) compared to the one-third-dose group (28.5%, *P* =.001). Both groups exhibited significant reductions in CRT, SRF thickness, and SFCT (*P* <.001), with no significant intergroup differences. Baseline CRT and fluorescein leakage patterns influenced treatment response.

**Conclusions:**

Both one-third-dose and half-dose PDT effectively improved visual and anatomical outcomes in patients with chronic CSC. However, half-dose PDT demonstrated superior efficacy in achieving SRF resolution and greater visual acuity gains with a lower recurrence rate.

**Clinical trial number:**

Not applicable.

## Introduction

Central serous chorioretinopathy (CSC) is a condition characterized by the detachment of the neurosensory retina, which may or may not involve the retinal pigment epithelium (RPE). In most cases, acute episodes of CSC are self-limiting and typically resolve within 3 to 6 months [1]. However, spontaneous resolution does not always occur, with 30–50% of patients experiencing recurrences. Additionally, around 5% of cases progress to chronic CSC, leading to damage in the outer nuclear layer, photoreceptors, and RPE, which can result in irreversible visual impairment [2–4].

Conventional laser photocoagulation, when applied to leakage sites located away from the fovea, can seal the leakage and expedite the resolution of subretinal fluid (SRF). However, this approach carries risks, including retinal pigment epithelium (RPE) damage, iatrogenic choroidal neovascularization (CNV), and potential visual loss [5]. More recently, subthreshold micropulse laser (SML) has emerged as an alternative treatment for CSC. This technique uses repetitive laser pulses delivered in short intervals, activating the RPE without causing damage to the RPE or photoreceptors [6]. Photodynamic therapy (PDT) with Verteporfin has demonstrated efficacy in managing both acute and chronic CSC. (7–8) Despite its effectiveness, complications such as RPE atrophy, RPE tears, secondary CNV, and choriocapillaris ischemia have been reported following PDT [9]. To reduce these adverse effects and optimize outcomes, modified PDT protocols have been explored. These include reducing the Verteporfin dose, shortening laser treatment duration, decreasing laser power, or adjusting the interval between Verteporfin injection and laser application [10–13]. Studies on half-dose PDT have consistently shown promising outcomes compared to full-dose PDT [10–13]. Additionally, some evidence suggests that using a 30% dose of Verteporfin may offer beneficial effects in both acute and chronic CSC, potentially comparable to the results of half-dose or full-dose PDT protocols [14–16]. 

The high cost of Verteporfin, coupled with a persistent global shortage of the drug (brand name Visudyne^®^) since May 2020, has significantly limited treatment options for various chorioretinal diseases, including CSC, choroidal hemangioma, and polypoidal choroidal vasculopathy [17]. Furthermore, the availability of new Verteporfin batches remains unpredictable, with irregular delivery schedules that often result in unequal distribution across different regions or countries [17]. In light of these challenges, this retrospective study aims to compare the outcomes of half-dose Verteporfin (3 mg/m²) PDT with one-third-dose Verteporfin (2 mg/m²) PDT for the management of chronic CSC in our region.

## Methods

This retrospective, non-randomized, consecutive case series was conducted in accordance with the Declaration of Helsinki to compare two different doses of Verteporfin (two PDT protocols) for the treatment of chronic CSC. The study population was recruited from Farvardin Eye Clinic and Poostich Eye Center, affiliated with Shiraz University of Medical Sciences, Shiraz, Iran, between March 2021 and May 2022. Institutional Review Board approval was obtained from Shiraz University of Medical Sciences, and the requirement for informed patient consent was waived due to the study’s retrospective design.

Patients were included in the study if they met the following criteria: [1] symptoms were present for the first time, with the episode duration exceeding 3 months, or medical records confirmed the presence of subretinal fluid (SRF) for more than 3 months, and the patient had been treated with either half-dose PDT or one-third-dose PDT; [2] age between 18 and 60 years; [3] evidence of SRF involving the macula detected via spectral-domain optical coherence tomography (SD-OCT); and [4] active fluorescein leakage observed during fluorescein angiography (FA).

Patients were excluded if they met any of the following criteria: [1] prior PDT, intravitreal injections of anti-vascular endothelial growth factor (VEGF) agents or steroids, or a history of intraocular surgery; [2] chronic cystic changes or significant retinal atrophic changes identified on OCT images; [3] evidence of other macular abnormalities, such as choroidal neovascularization (CNV) or polypoidal choroidal vasculopathy (PCV), detected via optical coherence tomography angiography (OCTA); [4] follow-up duration of less than 12 months or incomplete data; [5] poor image quality; [6] systemic diseases with potential ocular involvement; and [7] refractive error exceeding ± 6 diopters (spherical equivalent).

A total of 72 eyes from 72 patients with chronic CSC were included in the study. Due to a severe shortage of Verteporfin from March 2021 to August 2021, the first 21 consecutive patients underwent PDT using a one-third-dose (2 mg/m²) Verteporfin protocol (Visudyne^®^, Novartis AG, Basel, Switzerland). Following this period, as Verteporfin availability improved, the remaining 51 patients were treated using the half-dose (3 mg/m²) protocol. In cases of bilateral involvement, the right eye, which was treated first, was selected for the study.

The collected data included demographic information, PDT treatment spot size, best-corrected visual acuity (BCVA), central retinal thickness (CRT), subretinal fluid (SRF) thickness, and subfoveal choroidal thickness (SFCT) at baseline, as well as at 3 and 12 months post-treatment. Additional baseline assessments included the presence of pigment epithelial detachment (PED), intraretinal hyperreflective foci (HF), and the status of the foveal ellipsoid zone (EZ) on OCT. All enrolled patients underwent OCTA (Optovue Avanti-RTVue-XR, Optovue, Fremont, California, USA) to identify any signs of macular neovascularization (MNV).

Baseline evaluations also included fundus imaging, blue autofluorescence imaging, and fluorescein angiography (FA) using ultra-widefield scanning laser ophthalmoscopy (UWF-SLO) with the California device (Optos, Marlborough, MA, USA) to confirm the diagnosis and plan PDT treatment. Laser application was targeted at area(s) of active leakage identified on FA. The target area(s) is often set so that the diameter of this spot covers the hyperfluorescent area(s) corresponding point(s) of leakage on FA. Leakage patterns were classified as discrete or diffuse, and their location was categorized as confined to the foveal avascular zone (FAZ) or extending beyond it. Data were extracted from electronic medical records through the clinical information system.

PDT was performed by administering either a half-dose (3 mg/m²) or one-third-dose (2 mg/m²) of Verteporfin (Visudyne^®^, Novartis AG, Basel, Switzerland) under the supervision of (MK. J). Verteporfin was infused over 10 min, followed by laser application (689 nm wavelength, total light energy of 50 J/cm² for 83 s) to the area of angiographic leakage observed on FA, starting 5 min after infusion. Patients were instructed to avoid sunlight exposure for 48 h after treatment. Informed consent for PDT was obtained from all patients following a discussion of the procedure’s potential risks and benefits.

The primary outcome measures were improvements in BCVA and the resolution of SRF at 3- and 12-month follow-ups after PDT treatment. All patients underwent OCT scans (Spectralis^®^, Heidelberg Engineering, Heidelberg, Germany) prior to treatment and at 3 and 12 months post-treatment. For OCT imaging, a raster scan passing through the fovea was selected. Subfoveal choroidal thickness (SFCT) was manually measured from the outer border of the retinal pigment epithelium to the inner boundary of the suprachoroidal space. The measurements were performed at the foveal center using the digital calipers provided by the OCT software. Similarly, SRF thickness was measured at the central point using the same software.

The foveal ellipsoid zone (EZ) status was assessed and classified as intact, disrupted, or lost. Hyperreflective foci (HF) were defined as small, well-circumscribed, dot-like lesions with a reflectivity equal to or exceeding that of the RPE band. The relationships between EZ status, HF, and PED, and the remission rates in each treatment group, were analyzed.

Retreatment was considered if serous detachment of the neurosensory retina at the macula persisted or recurred during the follow-up period, but not earlier than 3 months after the initial treatment.

## Statistics

Statistical analyses were performed using IBM SPSS Statistics Version 23.0 (IBM Corp., Armonk, NY, USA). Continuous variables were presented as mean ± standard deviation (SD) with 95% confidence intervals (CIs). Normality of continuous variables was assessed using the Shapiro-Wilk test. Since the data did not follow a normal distribution, non-parametric tests (Wilcoxon signed-rank test and Mann–Whitney U test) were used for statistical comparisons. The Wilcoxon signed-rank test was used to compare baseline clinical data, with a p-value of less than 0.05 considered statistically significant. Comparisons between the two treatment groups were conducted using Fisher’s exact test and the Chi-square test for categorical variables, while the Mann–Whitney U test was applied for continuous data.The cumulative number of eyes achieving complete subretinal fluid (SRF) resolution and the recurrence of SRF were analyzed using Fisher’s exact test. A p-value of less than 0.05 was considered statistically significant for all analyses.

## Results

### Baseline demographic and clinical data

**Fig. 1:**
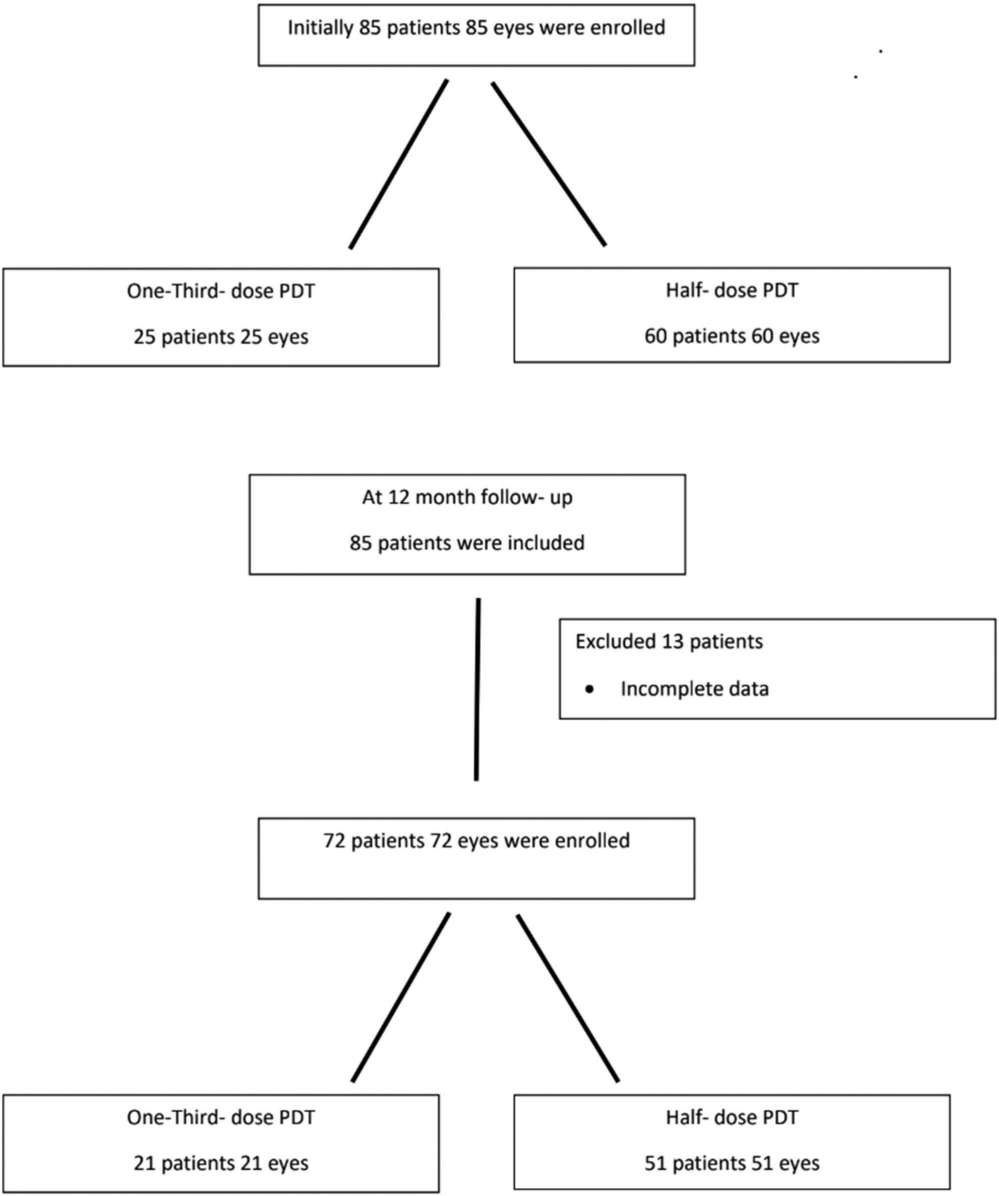
Patient Flowchart

A total of 85 eyes from 85 patients with chronic CSC were initially enrolled in the study. Of these, 25 eyes were treated with one-third-dose PDT, and 60 eyes received half-dose PDT. At the 12-month follow-up, data from 13 patients were incomplete, leaving 72 patients for analysis: 21 in the one-third-dose PDT group and 51 in the half-dose PDT group (Fig. 1).

Baseline demographic and clinical characteristics for the two treatment groups are summarized in Table 1. There were no significant differences between the groups in terms of mean age, sex distribution, duration of symptoms, history of systemic steroid use, mean BCVA, mean IOP, mean CRT, mean SRFT, mean SFCT, mean PDT spot size or the presence of pigment epithelial detachment, hyperreflective foci, and foveal ellipsoid zone disruption. Similarly, fluorescein angiography (FA) leakage pattern classifications did not differ significantly between the groups (Table 2).

**Table 1:** Baseline demographics and clinical data of the two treatment groups

	30%-Dose PDT Group	50%-Dose PDT Group	*P* value
	(*n* = 21)	(*n* = 51)	
Age, mean ± SD, years	44.90 ± 8.83	48.55 ± 1107	> 0.5
Gender (male/female)	17/4	40/10	> 0.5
Duration of symptoms, (mean ± SD), months)	8.5 ± 4.2	9.2.5 ± 6.2	> 0.5
History of systemic steroid use, yes number. (%)	3(14.28)	5(10)	> 0.5
IOP, mean ± SD, mm Hg	13.9 ± 2.6	14.3 ± 3.2	> 0.5
BCVA, mean ± SD (ETDRS letters)	72.4 ± 3.9	74.4 ± 4.2	> 0.5
CRT, mean ± SD, µm	365.7 ± 97.5	382.5 ± 131.2	> 0.5
SRFT, mean ± SD, µm	181.8 ± 89.9	203.5 ± 132.7	> 0.5
SRCT, mean ± SD, µm	242.9 ± 45.0	228.3 ± 48.9	> 0.5
Hyperreflective foci yes number. (%)	12(57.1)	28(54.9)	> 0.5
Pigment epithelial detachment, yes number. (%)	6(28.5)	13(25.4)	> 0.5
Ellipsoid zone disruption yes number. (%)	8(38.0)	16(31.3)	> 0.5
Mean ± SD PDT spot size (µm)	3200 ± 341	3300 ± 213	> 0.5

IOP; intra ocular pressure. BCVA; best corrected visual acuity. CRT; central retinal thickness. SRFT; subretinal fluid thickness

SRCT; sub foveal choroidal thickness. PDT; photodynamic therapy. SD; standard deviation

**Table 2:** Angiographic characteristics of the two treatments group

		30%-Dose PDT Group		50%-Dose PDT Group		*P* value
Location of leakages, no (%)						
limited to FAZ		6(28.5)		16(31.3)		> 0.05
Beyond the FAZ		3(14.2)		7(13.7)		> 0.05
Both		12(57.1)		28(54.9)		> 0.05
**Type of leakages**, **no(%)**						
Discrete		8(38)		16(31.3)		> 0.05
Diffused		13(62)		35(68.7)		> 0.05

PDT; photodynamic therapy. FAZ; foveal avascular zone

### Resolution of SRF

In the one-third-dose PDT group, complete resolution of SRF on OCT was observed in 14 eyes (66.6%) at 3 months and in 15 eyes (71.4%) at 12 months. In the half-dose PDT group, SRF resolved completely in 40 eyes (78.4%) at both 3 and 12 months. Recurrence of SRF occurred in 3 of 15 eyes (20%) in the one-third-dose group and in 3 of 40 eyes (7.5%) in the half-dose group within 12 months (Table 3).

**Table 3:** OCT imaging based complete SRF resolution and recurrence in the 2 treatment groups

	30%-Dose PDT Group	50%-Dose PDT Group	Difference Between	*P*-value
	(*n* = 21)	(*n* = 51)	2 Groups, % (95% CI)	
Complete resolution at 3 months n (%)	14 (66.6%)	40 (78.4%)	11.8	0.03
Complete resolution at 12 months n (%)	15(71.4%)	40(78.4%)	7	0.04
Recurrences of SRF at 12 months n (%)	3 from 15	3 from 40		
	20%	7.50%	12.5	0.03

PDT; photodynamic therapy

**Fig. 2:**
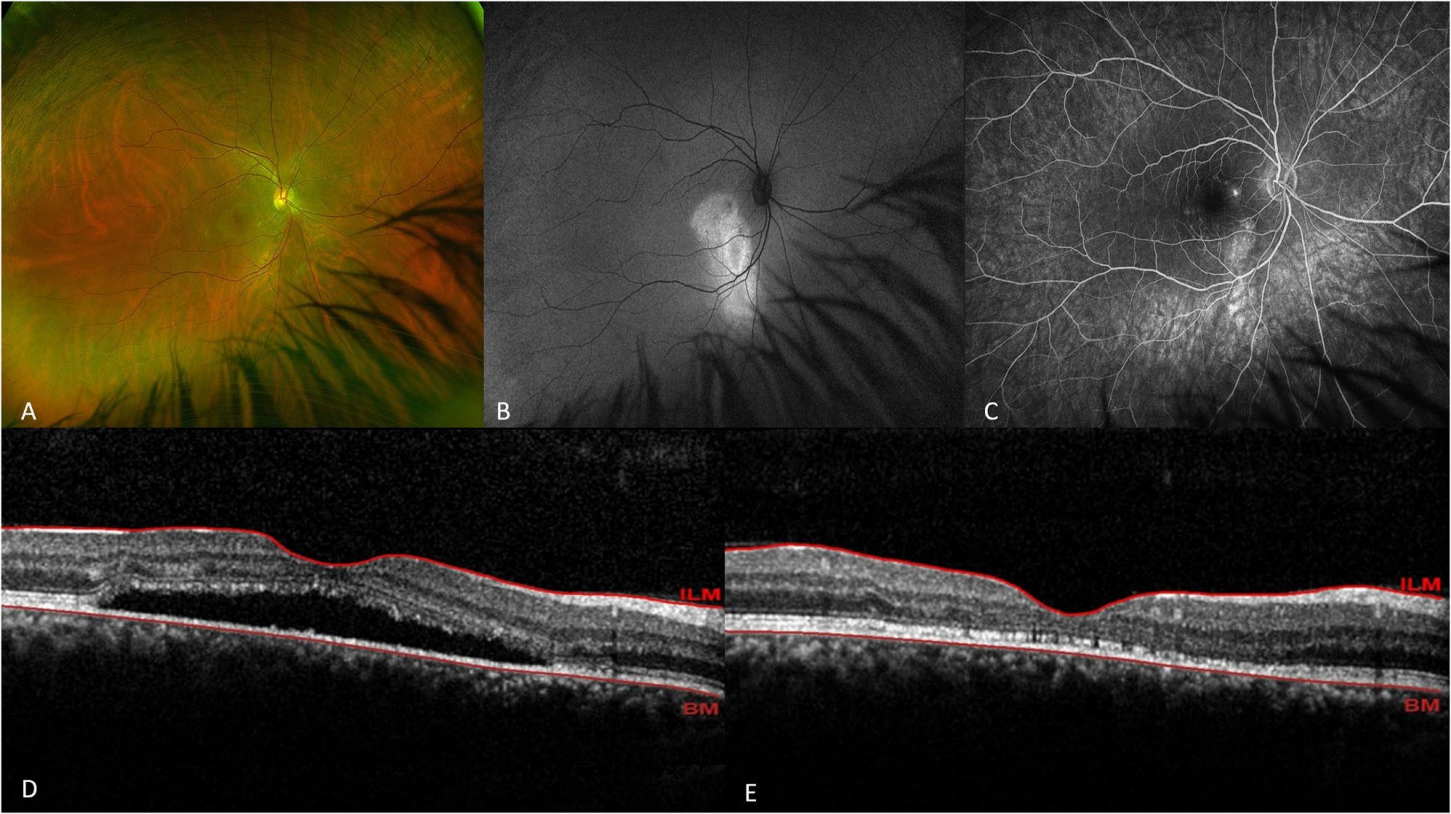
Optos fundus photograph (**A**), fundus autofluorescence (**B**), fluorescein angiography (**C**), and macular OCT before (**D**) and after (**E**) treatment in patient treated with half-dose PDT. The complete resolution of foveal subretinal fluid after PDT is evident

**Fig. 3:**
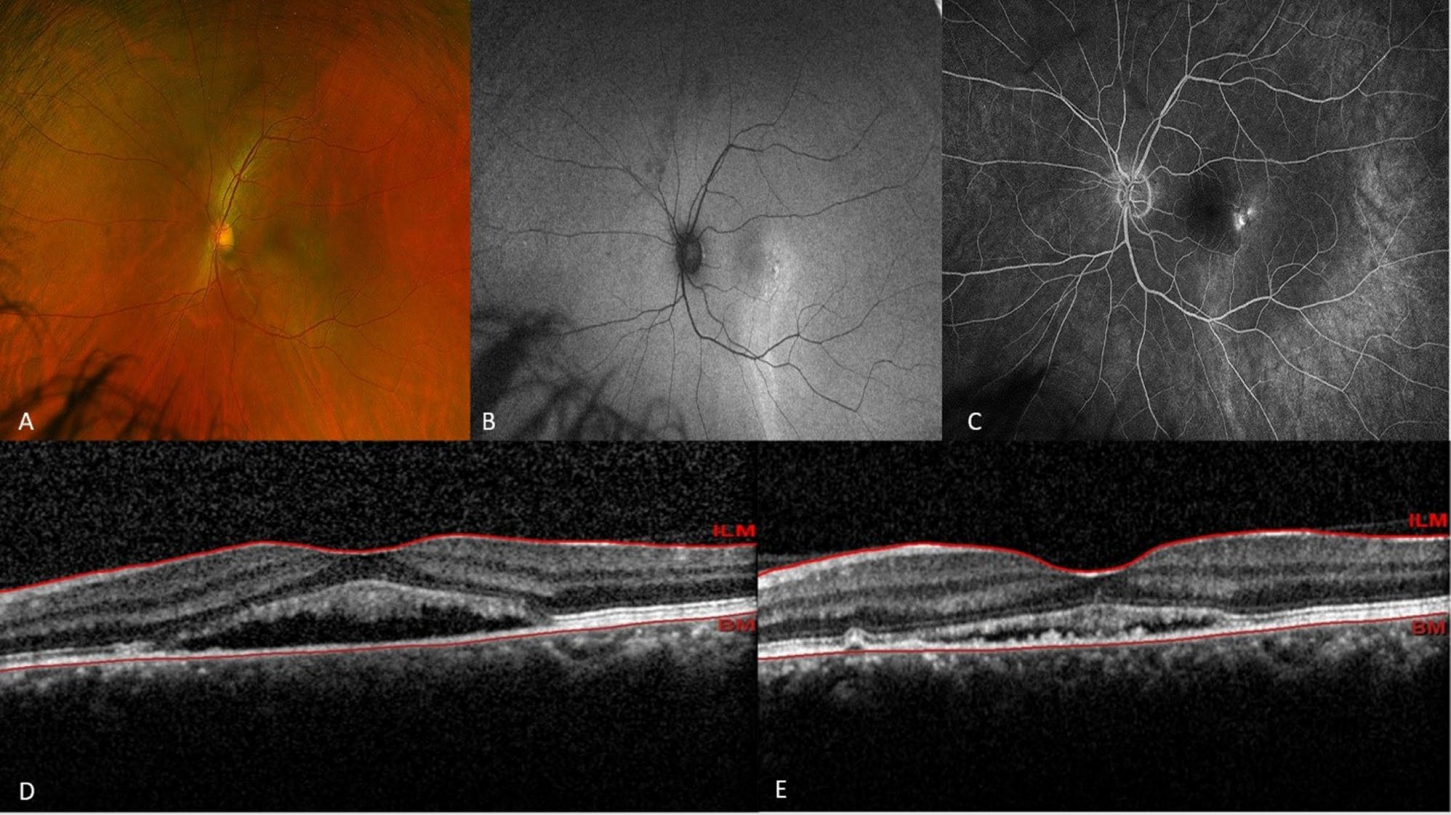
Optos fundus photograph (**A**), fundus autofluorescence (**B**), fluorescein angiography (**C**), and macular OCT before (**D**) and after (**E**) treatment in patient treated with one-third-dose PDT. The near complete resolution of foveal subretinal fluid after PDT is evident

The mean difference in the SRF resolution rates between the two groups was − 11.8% (95% CI: −19.4% to − 2.8%; *P* =.82, based on the asymptotic Wald test) at 3 months and − 7% (95% CI: −15.7% to − 2.1%; *P* =.72) at 12 months (Fig. 2 and 3).

Using the Pearson χ² test, the half-dose PDT group demonstrated significantly higher rates of SRF resolution compared to the one-third-dose group at both 3 and 12 months (*α* = 0.0125, *P* =.03; *α* = 0.0125, *P* =.04, respectively) (Table 3).

Twelve eyes (80%) in the one-third-dose group and 37 eyes (92.5%) in the half-dose group achieved complete SRF absorption without recurrence during the 12-month follow-up. A log-rank test revealed that the SRF recurrence rate at 12 months was significantly higher in the one-third-dose group compared to the half-dose group (20% vs. 7.5%; *P* =.015).

### Changes in visual acuity

At the 12-month follow-up after PDT, both treatment groups demonstrated a significant improvement in BCVA (*P* <.05) compared to baseline. In the one-third-dose group, the mean ± SD BCVA increased from an ETDRS letter score of 72.4 ± 3.9 at baseline to 77.1 ± 5.6 at 12 months, while in the half-dose group, the mean ± SD BCVA increased from 74.4 ± 4.2 at baseline to 80.2 ± 2.19 at 12 months. There were no significant differences between the two groups in terms of BCVA gain at the last follow-up (*P* =.32, paired *t*-test) (Table 4).

**Table 4:** Changes in the clinical data after PDT in the two treatment groups (At month-12)

	30%-Dose PDT Group	50%-Dose PDT Group	*P*-value
Mean ± SD gained BCVA (ETDRS letters)	4.70 ± 2.2	5.80 ± 2.1	0.32
Mean ± SD BCVA (ETDRS letters)	77.1 ± 5.6	80.2 ± 2.19	
Mean ± SD decreased in CRT(µm)	112.8 ± 19.5	111.1 ± 18.5	0.64
Mean ± SD CRT(µm)	252.9 ± 112.54	271.43 ± 131.51	
Mean ± SD decreased in SRFT(µm)	114 ± 12.4	109.8 ± 15.7	0.71
Mean ± SD SRFT(µm)	67.8 ± 111.6	93.63 ± 125.5	
Mean ± SD decreased in SFCT(µm)	43.8 ± 8.2	40 ± 6.9	0.31
Mean ± SD SFCT(µm)	199.1 ± 46.48	188.31 ± 34.34	

CRT; central retinal thickness, SRFT; subretinal fluid thickness, SFCT; sub foveal choroidal thickness

Compared to baseline, 6 eyes (28.5%) in the one-third-dose group gained 10 or more letters at 12 months, whereas 26 eyes (52.0%) in the half-dose group achieved a gain of 10 or more letters (*P* =.001, Fisher’s exact test). The mixed-effects model indicated that the improvement in BCVA was significantly greater in the half-dose group compared to the one-third-dose group (*P* <.05).

### Changes in central retinal thickness and subretinal fluid thickness

At 12 months following PDT, both groups exhibited a significant reduction in CRT and SRFT (*P* <.001 for both). In the one-third-dose group, the mean ± SD decrease in CRT was 112.8 ± 19.5 μm, while the mean ± SD decrease in SRFT was 114 ± 12.4 μm. In the half-dose group, the mean ± SD decrease in CRT was 112.8 ± 19.5 μm, and the mean ± SD decrease in SRFT was 111.1 ± 18.5 μm. There were no significant differences between the two groups regarding changes in CRT and SRFT at the 12-month follow-up (*P* >.05 for both, paired *t*-test) (Table 4).

### Changes in subfoveal choroidal thickness

At 12 months post-treatment, both groups demonstrated a significant reduction in SFCT (*P* <.05). In the one-third-dose group, the mean ± SD decrease in SFCT was 43.8 ± 8.2 μm, while in the half-dose group, the mean ± SD decrease was 40 ± 6.9 μm. No significant differences were found between the two groups in terms of changes in SFCT at the last follow-up (*P* >.05, paired *t*-test) (Table 4).

### Patterns of fluorescein leakage

In the one-third-dose PDT group, the fluorescein leakage pattern was diffuse in 62% of patients and confined to the foveal avascular zone in 28% of patients on FA images. In the half-dose PDT group, the fluorescein leakage pattern was diffuse in 68.7% and confined to the foveal avascular zone in 31.3% of patients. No significant differences were observed between the two groups in terms of FA leakage patterns (Table 2).

### Comparison of baseline data regarding complete response to PDT treatment

We analyzed data from all patients who underwent treatment and achieved complete resolution of subretinal fluid (SRF) over the 12-month follow-up period. Out of the 72 patients in the study, 55 patients had complete resolution of SRF, including 40 from the half-dose group and 15 from the one-third-dose group. Despite the half-dose group having more than twice the number of patients, nominal data were analyzed using Fisher’s exact test, and continuous data were analyzed with Welch’s t-test due to variance differences between the groups.

Regardless of the treatment dose, patients with lower baseline CRT demonstrated significantly better treatment responses (*P* =.001). Regarding fluorescein leakage characteristics, patients with discrete leakage confined to the foveal area showed significantly better outcomes (*P* =.001). A comparison of additional data and OCT findings is presented in Table 5.

**Table 5:** Comparison of baseline data regarding to complete response to PDT treatment

	complete resolution of SRF		
	yes *N* = 55	No (*n* = 17)	*P* value
Half-dose PDT(n)	40	11	-
One-third-dose PDT(n)	15	6	-
Mean ± SD Age(years)	46.44 ± 11.16	48.84 ± 9.78	0.14
Mean ± SD Baseline CRT(µm)	319.67 ± 51.7	448.59 ± 144.9	0.001
Mean ± SD Baseline SFCT(µm)	223.62 ± 40	243.31 ± 243.31	0.1
Discrete leakage in FA(%)	33%	67%	0.001
limited to FAZ leakage in FA(%)	45%	27%	0.001
Persent of HRF(%)	48%	52%	0.12
Present of PED(%)	45%	55%	0.17
Present of EZD(%)	42%	58%	0.21

PDT; photodynamic therapy, CRT; central retinal thickness, SFCT; sub foveal choroidal thickness, FAZ; foveal avascular zone, HRF; hyper reflective foci, PED; pigment epithelial detachment, EZD; ellipsoid zone disruption

### Safety

No systemic adverse events related to verteporfin infusion were observed in any of the patients. Additionally, no ocular side effects, including the development of CNV, were reported during the follow-up period.

## Discussion

PDT is commonly used to treat chronic central serous chorioretinopathy (CSC) because it helps restore the choroidal vasculature and reduces vascular hyperpermeability (7, 18–19, 24). PDT was initially introduced for the treatment of CSC using standard dosing protocols (6.0 mg/m², 50 J/cm²). When treatment for CSC is indicated, half-dose PDT is currently the treatment of choice for achieving rapid SRF resolution, a faster improvement in BCVA, and a decreased risk of recurrence compared to other available treatments [24]. Based on the data of the recently reported RCTs, half-dose PDT should also be considered the treatment of choice for chronic CSC [24]. The high cost and global shortage of verteporfin have restricted treatment options for this choroidal hyperpermeability disorder. Thus, determining the optimal PDT protocol that maintains treatment efficacy remains essential. In this retrospective study, we compared the functional and structural outcomes of one-third-dose and half-dose PDT for chronic CSC.

Uetani et al. [16] conducted PDT with verteporfin at half and one-third of the conventional dosage in 16 patients with chronic CSC. At 3 months of follow-up, SRF resolved in all eyes (100%) in the half-dose PDT group, while only two eyes (33%) in the one-third-dose group showed resolution. In our study, 40 out of 51 patients (78.4%) in the half-dose PDT group and 14 out of 21 patients (66.6%) in the one-third-dose PDT group achieved complete SRF resolution without complications. While Uetani’s study reported significant visual acuity improvement in the half-dose group at 3 months, no such improvement was observed in the one-third-dose group. In contrast, our study found that both treatment groups demonstrated significant improvement in best-corrected visual acuity (BCVA) compared to baseline at the last follow-up, although the mixed-effects model revealed that BCVA improvement was greater in the half-dose group.

Pichai et al. [15] found that SRF reabsorption rates were 72% for the half-dose PDT group and 60% for the one-third-dose group in patients with chronic CSC. A similar trend was observed in our study, with the half-dose PDT group showing a higher percentage of eyes with SRF reabsorption compared to the one-third-dose group, although this difference was not statistically significant. BCVA improvements in their study were comparable, with both PDT dosages effectively improving BCVA at 12 months. Notably, the half-dose PDT group exhibited faster BCVA improvement compared to the one-third-dose group. In our study, 52% of patients in the half-dose group and 28.5% in the one-third-dose group achieved a gain of 10 or more letters in BCVA.

Zhao et al. [4] conducted PDT with half and one-third of the conventional dosage in 117 patients with acute CSC, showing SRF absorption in 92.9% and 73.8% of patients at 6 months following half-dose and one-third-dose PDT, respectively. Their study reported better anatomic and visual outcomes than those observed in patients with chronic CSC, as seen in Uetani [16], Pichai [15], and our study. The retinal pigment epithelium (RPE) barrier and pump functions are likely weaker in chronic CSC, and a stronger suppression of choroidal vessels may be required for SRF resolution in these patients compared to those with acute CSC (20–21, 24).

The ellipsoid zone (EZ) is a key indicator of photoreceptor integrity, and its role in the recovery of retinal function in patients with chronic CSC has been explored in previous studies [22]. Matušková et al. [23] found that EZ damage was associated with the presence of pigment epithelial detachment (PED) at baseline, which was identified as a negative predictive factor for visual improvement. Similarly, Altinel et al. [24] highlighted an intact EZ and lower central retinal thickness (CRT) values as predictors of favorable responses to photodynamic therapy (PDT). In our study, while no statistically significant differences were found in terms of EZ disruption, hyperreflective foci, or PED between eyes with complete versus incomplete resolution of SRF, eyes that failed to respond to treatment were more likely to present with higher baseline mean CRT values and discrete leakage outside the foveal avascular zone (FAZ).

This study has several limitations. First, its retrospective design with consecutively enrolled patients resulted in unequal sample sizes between groups, which may have influenced the statistical power of the comparisons. Additionally, the relatively small number of cases in each group limits the generalizability of our findings. Routine indocyanine green angiography (ICGA) was not performed for diagnosis and treatment planning, which could have provided additional insights into choroidal vascular changes. Furthermore, functional assessments such as microperimetry or multifocal electroretinography were not conducted due to the unavailability of these devices.

Despite these limitations, the study provides valuable real-world evidence comparing two reduced-dose PDT protocols for chronic CSC. Future prospective, randomized studies with larger and more balanced sample sizes, as well as long-term follow-up incorporate survival analysis to provide a more comprehensive understanding of treatment response and long-term treatment durability are warranted to further validate our findings and optimize PDT dosing strategies for chronic CSC management.

## Conclusion

This study demonstrated that both one-third-dose and half-dose photodynamic therapy (PDT) are effective in improving visual and anatomical outcomes in patients with chronic central serous chorioretinopathy (CSC). However, half-dose PDT was associated with higher rates of subretinal fluid (SRF) resolution, greater visual acuity gains, and lower recurrence rates compared to one-third-dose PDT. Baseline factors such as central retinal thickness and fluorescein angiographic leakage patterns significantly influenced treatment outcomes, underscoring the importance of individualized treatment planning. While both protocols provide viable options for managing chronic CSC, half-dose PDT appears to offer superior efficacy with a comparable safety profile. Further prospective studies with larger sample sizes and advanced functional assessments are warranted to refine dosing strategies and optimize treatment outcomes.

## Data Availability

No datasets were generated or analysed during the current study.
